# Reduced seasonal coronavirus incidence in high‐risk population groups during the COVID‐19 pandemic

**DOI:** 10.1002/iid3.1342

**Published:** 2024-07-18

**Authors:** Aliisa Heiskanen, Yannick Galipeau, Julian Little, Marc‐André Langlois, Curtis L. Cooper

**Affiliations:** ^1^ School of Epidemiology and Public Health, Faculty of Medicine University of Ottawa Ottawa Ontario Canada; ^2^ Department of Biochemistry, Microbiology & Immunology, Faculty of Medicine University of Ottawa Ottawa Ontario Canada; ^3^ Centre for Infection, Immunity and Inflammation (CI3) University of Ottawa Ottawa Ontario Canada; ^4^ Ottawa Hospital Research Institute Ottawa Ontario Canada

**Keywords:** COVID‐19, incidence, SARS‐CoV‐2, seasonal coronavirus, seroprevalence

## Abstract

**Background:**

Epidemiological data on seasonal coronaviruses (sCoVs) may provide insight on transmission patterns and demographic factors that favor coronaviruses (CoVs) with greater disease severity. This study describes the incidence of CoVs in several high‐risk groups in Ottawa, Canada, from October 2020 to March 2022.

**Methods:**

Serological assays quantified IgG and IgM antibodies to SARS‐CoV‐2, HCoV‐OC43, HCoV‐NL63, HCoV‐HKU1, and HCoV‐229E. Incident infections were compared between four population groups: individuals exposed to children, transit users, immunocompromised, and controls. Associations between antibody prevalence indicative of natural infection and demographic variables were assessed using regression analyses.

**Results:**

Transit users and those exposed to children were at no greater risk of infection compared to the control group. Fewer infections were detected in the immunocompromised group (*p* = .03). SARS‐CoV‐2 seroprevalence was greater in individuals with low income and within ethnic minorities.

**Conclusions:**

Our findings suggest that nonpharmaceutical interventions intended to reduce SAR‐CoV‐2 transmission protected populations at high risk of exposure. The re‐emergence of sCoVs and other common respiratory viruses alongside SARS‐CoV‐2 may alter infection patterns and increase the risk in vulnerable populations.

## Graphical abstract

Understanding seasonal coronavirus activity may provide valuable insights into what can be anticipated of SARS‐CoV‐2 as we move to an endemic era. Incident seasonal coronavirus infections were found to be similar between those exposed to children, transit users and a control group while fewer natural infections were identified in immunocompromised individuals. SARS‐CoV‐2 seroprevalence was found to be greater in individuals with low‐income and ethnic minorities using regression analyses.

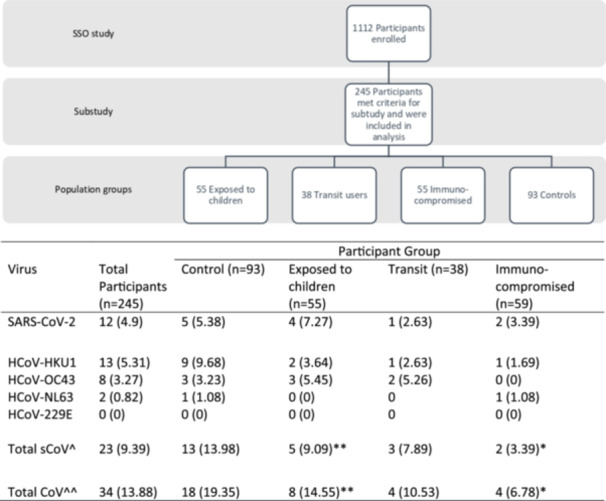

## 1 INTRODUCTION

The impact of SARS‐CoV‐2 has been experienced worldwide, resulting in over 6.9 million deaths.^1^ The pandemic has focused attention on the antigenically diverse coronavirus family. Seasonal coronaviruses (sCoVs) typically circulate at high levels in the community and are associated with 15%–30% of common cold cases.^2^ Understanding the epidemiological data related to sCoVs may help identify factors that favor infection and inform surveillance and control strategies.^3^

The relatively high recombination and mutation rate of human coronaviruses (HCoVs) has resulted in great genetic diversity and unpredictable changes in virulence.^4^ The emergence of cross‐species transmission events observed with SARS‐CoV‐1, MERS‐CoV, and SARS‐CoV‐2 has shown that CoVs represent an ongoing threat of emergence from multiple existing animal reservoirs. Humans are routinely infected by two genera of coronaviruses: alphacoronaviruses (HCoV‐229E and HCoV‐NL63) and betacoronaviruses (HCoV‐OC43, HCoV‐HKU1, SARS‐CoV‐1, MERS‐CoV, and SARS‐CoV‐2). Seasonal coronaviruses (HCoV‐OC43, HCoV‐NL63, HCoV‐229E, and HCoV‐HKU1) are omnipresent and typically circulate in the winter months. Reinfections are a common characteristic of seasonal coronaviruses, where adults are typically infected every 2–3 years, and reinfection can occur within months of initial infection.^5^, ^6^ Typical symptoms of sCoVs include rhinitis, headache, fever, and cough. Severe diseases and lower respiratory tract illness, such as pneumonia and bronchitis, are rare but more likely to occur in children, elderly, and immunocompromised individuals.^4^

Pre‐existing immunity to SARS‐CoV‐2 from previous seasonal coronavirus infection has been reported.^7^, ^8^, ^9^ However, the translation of neutralizing capabilities and durable cross‐immunity between sCoVs and pandemic coronaviruses is not fully understood. Pre‐existing immunity from sCoVs in the form of antibodies or B and T cell memory may enhance immune response to SARS‐CoV‐2 and reduce COVID‐19 severity.^7^, ^10^ Correlates of immunity are further complicated by cross‐reactivity among coronaviruses with antigenically related epitopes. Prior sCoV exposures may negatively impact the immune response to SARS‐CoV‐2 infection. Boosted antibodies may not be associated with protection or neutralization and have reduced affinity towards the novel SARS‐CoV‐2 virus. This suggests immune imprinting or original antigenic sin (OAS) and has been associated with negative clinical outcomes.^11^, ^12^, ^13^ The relationship between SARS‐CoV‐2 and sCoV antibodies and immunity is unclear but does impact vaccination strategies and disease outcomes.

SARS‐CoV‐2 seroprevalence is highly variable among studies and is dependent on demographic characteristics, variant of concern, assay type, and public health measures in place at the time of evaluation. Ottawa, Canada, is an economically affluent city and the headquarters of multiple federal government departments and home of multiple technology companies. Almost 20% of citizens used public transit before the COVID‐19 pandemic, placing it fourth among Canadian cities for proportion of usage.^14^ The culturally and linguistically diverse population, with high reliance on public transit, makes Ottawa an ideal location to assess respiratory virus infection and immunity. As in most other settings, the SARS‐CoV‐2 wild‐type was the primary local variant until March 2021. Thereafter, the alpha variant, and to a lesser extent, the delta variant became prominent in Ontario.^15^ Key public health measures in place during the study period included closure or reduced capacity in public areas, limits on close contacts, stay‐at‐home orders, masking, utilization of remote school modalities and reduced class sizes, vaccination requirements, and travel restrictions, which all impacted population exposure risk.

Understanding sCoV epidemiology can serve as an informative model for what may be expected of SARS‐CoV‐2 or other emerging CoVs in a post‐pandemic setting. Identifying demographic groups and factors associated with infection can be used to inform intervention methods and protect vulnerable populations. This study aimed to describe the incidence of SARS‐CoV‐2 and seasonal coronavirus infection in several high‐risk groups in Ottawa, Ontario, from October 2020 to March 2022. We also aimed to identify demographic factors associated with coronavirus antibody prevalence acquired from natural infection.

## 2 METHODS

**Figure 1:**
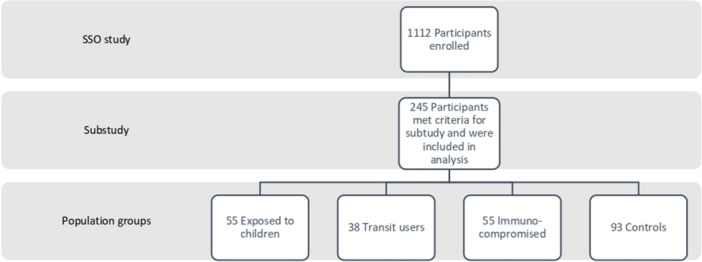
Flow diagram of participants selected from Stop the Spread Ottawa study for the subgroup analysis presented in this article.

Study participants were enrolled in the Stop the Spread Ottawa Study (SSO) described elsewhere.^16^, ^17^ SSO is approved by the Ottawa Health Science Network Research Ethics Board (#2020‐0481‐01H). Of the 1112 participants enrolled in the initial SSO study, 245 were selected for this cross‐sectional nested cohort analysis (Figure 1). Participants were prioritized for inclusion if they had plasma samples available from three separate time points: baseline, Month 3, and Month 10. If samples were unavailable from these time points, the sample collected closest to the time point of interest was selected. All visits occurred between October 22, 2020 and February 28, 2022. Covariates suspected to influence SARS‐CoV‐2 seroprevalence were determined a priori. Missing demographic variables from participant questionnaires were verified using medical records where available and by participant call‐back. The main outcome of interest was incident HCoV‐OC43, HCoV‐NL63, HCoV‐229E, HCoV‐HKU1, and SARS‐CoV‐2 infections (Supporting Information S1: Appendix Table 1). Incident infections were compared between population groups of interest. We also assessed associations between demographic characteristics and antibody prevalence. Seasonal coronavirus IgG titers were used to assess associations with demographic variables because all participants were seropositive at baseline. Serology methodology, infection definitions, and cutoff points can be found in Section 2.2.

### 2.1 Population groups

Participants were selected for this study if they met the criteria for one of three population groups of interest: high level of exposure to children at home or in the workplace, transit users or those that work in the public transportation industry, or immunocompromised individuals. Participants were categorized into the group exposed to children if they worked in a school or daycare or lived with one or more children in their household. Participants were classified in the transit user group they used public transportation at least once a week on average across follow‐up or worked in the public transportation industry (i.e., city bus operator, lightrail operator, and taxi driver). Participants were classified as immunocompromised if they had a primary or secondary immunodeficiency or required immunomodulatory treatment incorporating a range of conditions such as multiple sclerosis, rheumatoid arthritis, cancer, diabetes, HIV, asthma or COPD requiring medication, and organ/bone marrow transplant recipients. A control group of healthy individuals with minimal to no exposure to transit or children was included. Participants who met inclusion criteria for more than one group were categorized into the group with greater hypothesized exposure. Those exposed to children and transit users were hypothesized to be at greater risk of exposure compared to the control group. Children are frequently identified as the index case of respiratory disease in households and are known to be the primary drivers of viral transmission in the community, increasing exposure in this group.^18^, ^19^ Use of or work in the public transportation industry involves prolonged social contact in an enclosed space which has historically been assumed to increase the risk of respiratory virus transmission.^20^ If a participant was both exposed to children and a transit user, they were categorized as a transit user. Immunocompromised individuals were hypothesized to be at reduced risk as we assumed they would take more precautions to avoid exposure. Sensitivity analyses were conducted removing participants that met criteria for more than one group.

### 2.2 Laboratory methods

Immunoglobulin G (IgG) titers (BAU/mL) against SARS‐CoV‐2 spike (S), receptor binding domain (RBD), and nucleocapsid (N) proteins in participant serum were quantified using a high throughput chemiluminescent direct ELISA as described before.^21^ Incident COVID‐19 infections were identified through serology and self‐report of positive PCR and rapid antigen test (RAT). Self‐reported positive results were validated using medical records where possible. Natural immunity was defined if a participant was positive (signal‐to‐cut‐off [SCO] > 1) for anti‐N IgG and either anti‐S or anti‐RBD IgG. Thresholds were established at a rate of 3% false discover rate (FDR) from the density distribution of pre‐pandemic sera. In the event of indiscriminate positive serum samples for those with no self‐reported test results, natural immunity was determined by examining the signal strength (magnitude of SCO) and the preceding and subsequent serum results.^17^ Participants with an infection confirmed by PCR or positive serology before baseline visit were considered SARS‐CoV‐2 seropositive at baseline.

Plasma IgG and IgM titers (relative luminescent units [RLUs]) against HCoV‐OC43, HCoV‐HKU1, HCoV‐229E, and HCoV‐NL63 spike proteins were quantified using a chemiluminescent ELISA modified from previously described assays.^21^, ^22^ Both IgG and IgM were measured at a single dilution and data was scaled to account for inter‐plate variability. The distribution of fold changes for each sCoV was evaluated separately. It was assumed that in most cases, there would be limited CoV infection during the period of evaluation, so any changes in antibody titer caused by infection would appear as an outlier.^6^ A twofold increase in titer between paired samples was used to define IgG seroconversion. Acute infection was defined if the IgM titer was greater than the mean +(3SD/2SD). Examination of IgG and IgM titers over time was used to verify infection in all instances where either IgG or IgM seroconversion was observed. Serology results where a participant tested positive for more than one seasonal coronavirus at a single time point were assumed to indicate cross‐reactivity rather than a co‐infection. It was assumed the infection was caused by the most prevalent seasonal coronavirus in the Ottawa region. Infections were classified using the following hierarchal ranking system from highest to lowest expected prevalence: OC43 > NL63 > HKU1 > 229E.^23^, ^24^, ^25^, ^26^

### 2.3 Statistical analysis

Categorical or dichotomous variables were presented as proportions. Continuous variables with a normal distribution were presented as means with standard deviation. Normality of data was assessed using Q‐Q plots and histograms. Linearity was assessed through visual inspection of scatter plots. Differences between categorical or dichotomous variables were assessed using chi‐square tests. Differences between continuous variables were assessed using Student's *t* test. The number of incident infections was compared between groups using the chi‐square test and Fisher's exact test for small cell sizes. The groups exposed to children and public transit were combined and classified as a high‐risk group where sample size was a concern. SARS‐CoV‐2 seropositivity resulting from natural infection was used as the dependent variable in logistic regression. HCoV‐NL63, HCoV‐OC43, HCoV‐HKU1, and HCoV‐229E IgG antibody titers were used as dependent variables in generalized linear models. A univariate regression analysis was performed as part of an exploratory analysis for all variables to determine associations between SARS‐CoV‐2 seroprevalence and sCoV antibody titers and demographic variables. Variables that were significant using a cutoff of 0.2 or were determined clinically important a priori were added to a multivariable model. Likelihood ratio tests were used to assess the fit of nested models. Logistic regression model fit was evaluated using the Hosmer–Lemeshow test and ROC statistics. Results were presented as odds ratios (ORs) and 95% confidence intervals (CIs). Residual plots were used to assess the model fit of linear models. Variable estimates and *p* values were presented. Sensitivity analyses were conducted removing participants that fit the criteria for more than one population group. The significance level was defined at *p* < .05. All analyses were conducted using SAS Analytics Software.

## 3 RESULTS

### 3.1 Participant characteristics

Two‐hundred forty‐five participants were included in the analysis. Fifty‐five were identified as exposed to children, 38 were classified as transit users, 59 were immunocompromised, and 93 served as controls. The demographic characteristics of all participants are reported in Table 1. Across all participants, the average age was 50 years, 54.7% were women, 91.0% were white, and 63.6% had at least a university degree. A comparison of demographic characteristics between each population group of interest and the control group revealed transit users and those exposed to children to be younger (*p* < .01, *p* < .01), and that those exposed to children have a higher household income (*p* < .01). The immunocompromised group was older (*p* = .02) and more likely to be male compared to the control group (*p* = .02).

**Table 1:** Characteristics of 245 participants stratified into population groups of interest.

		Population group				
Characteristic	All participants (*n* = 245)	Exposed to children (*n* = 55)	Transit user (*n* = 38)	Immuno‐compromised (*n* = 59)	Control (*n* = 93)	*p* Value
Sex (*n*, %)						
Female	134 (54.69)	34 (61.82)	23 (60.53)	23 (38.98)	54 (58.06)	.047
Male	111 (45.31)	21 (38.18)	15 (39.47)	36 (61.02)	39 (41.94)	
Age (mean, SD)	50 (14.21)	46 (9.15)	42 (14.06)	57 (12.18)	51 (15.71)	<.001
Income (*n*, %)						
<$59,999	40 (16.33)	3 (5.45)	8 (21.05)	16 (27.12)	13 (13.98)	<.001
$60,000–89,999	44 (17.96)	3 (5.45)	9 (23.68)	14 (23.73)	18 (19.35)	
$90,000–119,999	43 (17.55)	14 (25.45)	5 (13.16)	11 (20)	13 (13.98)	
>$120,000	93 (37.96)	34 (61.82)	13 (34.21)	18 (19.35)	34 (36.56)	
No response	25 (10.20)	1 (1.82)	3 (7.89)	6 (10.17)	15 (16.13)	
Ethnicity (*n*, %)						
White	223 (91.02)	50 (90.91)	34 (89.47)	51 (86.44)	88 (94.62)	.214^b^
Other^a^	22 (8.98)	5 (9.10)	4 (10.53)	8 (13.56)	5 (5.38)	
Education (*n*, %)						
Highschool or less	20 (8.16)	3 (5.45)	6 (15.79)	4 (6.78)	7 (7.53)	.557^b^
Trade/college	62 (25.31)	10 (18.18)	9 (23.68)	20 (33.90)	23 (24.73)	
Bachelor's degree	100 (40.82)	26 (47.27)	12 (31.58)	22 (37.29)	40 (43.01)	
Graduate or higher	56 (22.86)	16 (29.09)	10 (26.32)	10 (16.95)	20 (21.51)	
No response	7 (2.86)	0	1 (2.7)	3 (5.45)	3 (2.94)	
Smoking status (*n*, %)						
Current smokers	14 (5.71)	2 (3.64)	3 (7.89)	6 (10.17)	3 (3.23)	.216^b^
Non‐smokers	231 (94.29)	53 (96.36)	35 (92.11)	53 (89.83)	90 (96.77)	

*Note*: Continuous variables are presented as mean (SD) and categorical variables presented as *N* (%). Differences between groups were assessed using Student's *t* tests for continuous variables and chi‐square tests for categorical variables.

^a^ Ethnicity was classified as either white or other due to small number of nonwhite participants. The “other” category included the following ethnicities: South Asian (*n* = 5), West Asian (*n* = 4), Filipino (*n* = 2), Latin American (*n* = 2), Indigenous (*n* = 2), Black (*n* = 1), Chinese (*n* = 1), and Other (*n* = 5).

^b^ Groups exposed to children and transit users combined to account for small cell sizes.

### 3.2 Incident infections

Among the 245 participants, 34 (13.9%) had antibody titers to SARS‐COV‐2, HCoV‐OC43, HCoV‐NL63, HCoV‐E229, or HCoV‐HKU1 over the 16‐month follow‐up period suggestive of recent infection. Incident SARS‐CoV‐2 infections were identified in 12 (4.9%) participants, and 23 (9.4%) participants were recently infected by at least one sCoV (Table 2). HCoV‐HKU1 was the most common sCoV identified (proportion with an infection: 5.3% [*n* = 13]), followed by HCoV‐OC43 (3.3% [n = 8]), HCoV‐NL63 (0.8% [n = 2]), and HCoV‐229E (no infections detected). Among the population groups of interest, the proportion with sCoV infection was greatest among the control group (14.0% [*n* = 13]), followed by those exposed to children (9.1% [*n* = 5]), transit users (7.9% [*n* = 3]), and immunocompromised participants (3.4% [*n* = 2]) (Table 2). The proportion of participants with sCoV infection(s) did not differ between those exposed to children, transit users, and our control group. Considerably fewer infections were observed in those with an immune compromising condition compared to the control group (*p* = .03). At baseline, all 245 participants had elevated plasma IgG for HCoV‐229E, HCoV‐NL63, HCoV‐HKU1, and HCoV‐OC43 and 70 (28.6%) of participants were seropositive for SARS‐CoV‐2, denoting a prior infection. The control group had the greatest number of SARS‐CoV‐2 anti‐N seropositive participants (44.0% [*n* = 41]), followed by those exposed to children (30.9% [*n* = 17]), transit users (18.4% [n = 7]) and immunocompromised individuals (8.5% [*n* = 5]) (Table 3).

**Table 2:** Number of coronavirus infections detected among 245 participants from October 22, 2020, to February 28, 2022 stratified by population group.

		Participant group			
Virus	Total participants (*n* = 245)	Control (*n* = 93)	Exposed to children (*n* = 55)	Transit (*n* = 38)	Immuno‐compromised (*n* = 59)
SARS‐CoV‐2	12 (4.9)	5 (5.38)	4 (7.27)	1 (2.63)	2 (3.39)
HCoV‐HKU1	13 (5.31)	9 (9.68)	2 (3.64)	1 (2.63)	1 (1.69)
HCoV‐OC43	8 (3.27)	3 (3.23)	3 (5.45)	2 (5.26)	0 (0)
HCoV‐NL63	2 (0.82)	1 (1.08)	0 (0)	0	1 (1.08)
HCoV‐229E	0 (0)	0 (0)	0 (0)	0	0 (0)
Total sCoV^b^	23 (9.39)	13 (13.98)	5 (9.09)^a^	3 (7.89)	2 (3.39)*
Total CoV^c^	34 (13.88)	18 (19.35)	8 (14.55)^a^	4 (10.53)	4 (6.78)*

*Note*: Comparisons between those exposed to children, transit users, and immunocompromised groups and the control group were conducted using Fisher's exact test. Results are presented as the number of participants infected by each virus (*N*) and the proportion of the group infected (%).

* Significant at *p* < .05.

^a^ Chi‐square test used.

^b^ Number (%) of participants that tested positive for HCoV‐NL63, HCoV‐OC43, HCoV‐HKU1, or HCoV‐229E.

^c^ Number (%) of participants that tested positive for HCoV‐NL63, HCoV‐OC43, HCoV‐HKU1, HCoV‐OC43, and SARS‐CoV‐2. A participant in the group with exposure to children tested positive for SARS‐CoV‐2 and HCoV‐OC43, so sCoV and SARS‐CoV‐2 infections do not sum to the total in the “Total Participants” and “Exposed to children” columns.

**Table 3:** Demographic variables of 245 participants stratified by SARS‐CoV‐2 seropositivity status indicative of prior natural infection.

Variable	SARS‐CoV‐2 Seropositive (*n* = 70)	SARS‐CoV‐2 Seronegative (*n* = 175)	OR	95% CI
Population group				
Exposed to children	17/55 (30.90)	38/55 (69.09)	0.81	0.35–1.86
Transit user	7/38 (18.42)	31/38 (81.58)	**0.35**	**0.13–0.96**
Immunocompromised	5/59 (8.47)	54/59 (91.53)	**0.05**	**0.02–0.17**
Control	41/93 (44.09)	52/93 (55.91)	Ref	Ref
Sex				
Male	34/111 (30.63)	77/111 (69.37)	Ref	Ref
Female	36/134 (26.87)	98/134 (73.13)	0.58	0.29–1.16
Ethnicity				
White	59/223 (26.46)	164/223 (73.54)	Ref	Ref
Other	11/22 (50.0)	11/22 (50.0)	**6.73**	**2.06–21.94**
Age	53.04 (SD: 15.29)	48.77 (13.60)	**1.04**	**1.02–1.07**
Smoking status				
Smoker	1/14 (7.14)	13/14 (92.86)	0.25	0.03–2.38
Nonsmoker	69/231 (29.87)	162/231 (70.13)	Ref	Ref
Education				
Highschool or less	7/27 (25.93)	20/27 (74.07)	0.77	0.21–2.78
Trade/college	13/62 (20.97)	49/62 (79.03)	0.59	0.22–1.58
Bachelor's degree	34/100 (34.0)	66/100 (66.0)	1.24	0.53–2.89
Masters’ or higher	16/56 (28.57)	40/56 (71.43)	Ref	Ref
Income				
<$59,999	13/20 (65.0)	7/20 (35.0)	**3.14**	**1.06–9.27**
$60,000–$89,999	10/44 (22.72)	34/44 (77.27)	0.96	0.35–2.64
$90,000–$120,000	8/43 (18.60)	35/43 (81.40)	0.76	0.27–2.10
+$120,000	28/93 (30.11)	65/93 (69.89)	Ref	Ref

*Note*: Continuous variables are presented as mean (SD), and categorical variables are presented as the number of seropositive participants divided by the number of participants in that group (%). Multivariate logistic regression was used to assess associations between SARS‐CoV‐2 seroprevalence and demographic variables at baseline, presented as odds ratios (ORs) and 95% confidence intervals (CIs). Significant associations are highlighted.

### 3.3 Factors associated with natural infection

SARS‐CoV‐2 seropositivity suggestive of natural infection was greater in older participants, those with a household income less than $59,999 and those of ethnic minority in a multivariable logistic regression analysis (Table 3). The odds of seropositivity was no greater in those exposed to children compared to our control group. Immunocompromised individuals were less likely to have anti‐N SARS‐CoV‐2 antibodies at baseline (OR: 0.05; 95% CI: 0.02–0.17) compared to our control group. Increasing age had a small positive association with SARS‐CoV‐2 antibody prevalence (OR: 1.04; 95% CI: 1.02–1.07). The odds of seropositivity was more than six times greater in those of ethnic minority compared to white participants (OR: 6.73, 95% CI: 2.06–21.94). The odds of seropositivity increased threefold in those with a household income less than $59,999 compared to those reporting a household income greater than $120,000 (OR: 3.14; 95% CI: 1.06–9.27). Increasing number of children in the household and level of public transportation use were not associated with SARS‐CoV‐2 seroprevalence (Supporting Information S1: Appendix Table 2).

Few associations were found between anti‐sCoV IgG antibody titers and demographic characteristics in multivariate linear regression models adjusted for age, sex, smoking status, income, education, and ethnicity (Table 4). Of consequence, smokers were found to have higher anti‐HCoV‐HKU1 antibody titers compared to non‐smokers (*p* = .03). Those that used public transportation daily or worked in the transportation industry were found to have higher anti‐HCoV‐HKU1 antibody titers compared to those that did not use public transit but this result was not consistent across the four sCoVs. Sensitivity analyses excluding participants that may have been categorized in more than one population group (*n* = 28) showed no appreciable change in results, with limited changes in *p* values and magnitude of effect (Supporting Information S1: Appendix Table 3).

**Table 4:** Demographic characteristics associated with coronavirus antibody titers for 245 participants at baseline.

	HKU1		OC43		NL63		229E	
Variable	Estimate	*p* Value	Estimate	*p* Value	Estimate	*p* Value	Estimate	*p* Value
Population group								
Control	Ref	Ref	Ref	Ref	Ref	Ref	Ref	Ref
Exposed to children	0.06	.38	0.01	.89	−0.01	.99	0.03	.77
Transit Users	0.10	.17	0.02	.85	−0.08	.35	−0.06	.48
Immunocompromised	−0.08	.20	−0.13	.13	−0.06	.42	−0.08	.34
Male	Ref	Ref	Ref	Ref	Ref	Ref	Ref	Ref
Female	0.05	.80	<0.01	.95	0.02	.72	−0.07	.25
Age	0.001	.53	<0.01	.80	<−0.01	.88	<−0.01	.28
Nonsmoker	Ref	Ref	Ref	Ref	Ref	Ref	Ref	Ref
Current smoker	0.11	**.03**	0.05	.71	−0.20	.12	−0.15	.26
Household income								
+$120,000	Ref	Ref	Ref	Ref	Ref	Ref	Ref	Ref
<$59,999	−0.14	.06	−0.05	.65	−0.03	.72	0.01	.91
$60,000–$89,999	−0.03	.69	−0.08	.42	−0.07	.40	−0.11	.20
$90,000–$120,000	−0.02	.76	−0.10	.30	−0.09	.31	−0.03	.74
Education								
Masters or higher	Ref	Ref	Ref	Ref	Ref	Ref	Ref	Ref
Highschool or less	−0.04	.66	−0.06	.64	−0.16	.19	−0.18	.14
Trade/college	−0.06	.39	−0.10	.31	−0.06	.50	0.04	.67
Bachelor's degree	<−0.01	.99	0.03	.73	−0.07	.38	−0.11	.15
White ethnicity	Ref	Ref	Ref	Ref	Ref	Ref	Ref	Ref
Nonwhite ethnicity	0.08	.34	0.18	.12	0.08	.45	0.03	.72

*Note*: Multivariate linear regression analysis was used to assess associations. Separate models were used for each virus. Results significant at the *p* < .05 level are highlighted.

## 4 DISCUSSION

The proportion of the study population with incident sCoV infections was 9.4%. Similar values were reported in the Ottawa region by the Eastern Ontario Regional Laboratory Association regional reference laboratory lab, where sCoV percent positivity ranged from 0% to 10% from October 2020 to December 2021.^27^ Given the ubiquitous nature of seasonal coronaviruses and high frequency of reinfections,^5^ under normal circumstances it is expected that most participants would experience a sCoV infection within the follow‐up period. A dramatic decline in common respiratory viruses, including influenza, RSV, parainfluenza viruses, rhinovirus, and sCoVs, was observed worldwide following the introduction of SARS‐COV‐2.^28^, ^29^, ^30^ This phenomenon has been partially attributed to public health‐mandated nonpharmaceutical COVID‐19 interventions initiated to slow SARS‐CoV‐2 transmission. These measures appear to have been effective in reducing sCoV transmission as well, contributing to the low number of incident infections observed in this observation period.

While children have been documented to have lower prevalence and severity of COVID‐19,^31^, ^32^ they still play an important role in SARS‐CoV‐2 transmission. Hundreds of students mix in an often crowded and poorly ventilated indoor school environment facilitating transmission.^33^ Additionally, children are more likely to have mild or asymptomatic infections and unknowingly propagate outbreaks among households and communities.^32^ In contrast to our analysis, a higher risk of SARS‐CoV‐2 infection has been reported in adults living with children in the United States^34^ and the United Kingdom.^35^ Other studies in North America have found that SARS‐CoV‐2 infection was no greater among school staff than in the community and that most COVID‐19 cases among students and teachers were acquired outside the school environment.^36^, ^37^, ^38^ One of these studies was conducted in Ontario during the first wave of the pandemic.^37^ As with our cohort, participants in this study were subject to similar community‐based nonpharmaceutical public health interventions so we believe it is appropriate to compare results between studies. A possible explanation for the discrepancy between the above‐mentioned studies is that Ontario schools were closed for a total of 20 weeks (March 14 to May 15, 2021), substantially longer than in any other Canadian province or territory.^39^ Intermittent school closures and shifts to online learning likely reduced exposure in this group and contributed to the reduced number of infections detected in SSO. Pre‐existing immunity caused by frequent respiratory infections has been proposed as a mechanism to explain lower susceptibility to SARS‐CoV‐2 infection in children.^35^, ^40^ A similar mechanism may explain why adults exposed to children experienced fewer SARS‐CoV‐2 infections. Adults living with children have been shown to experience more frequent respiratory infections than those who do not live with children, which may provide a greater degree of immune‐mediated protection against SARS‐CoV‐2.^41^

The proportion of transit users with CoV infections was comparable to our control group despite the hypothesized increased risk of exposure. The small number of CoV infections detected in transit users may be a consequence of effective public health measures implemented at the start of the pandemic to protect both transit users and operators. This included frequent cleaning and disinfection of facilities and high‐touch surfaces, limited capacity, and installation of physical barriers.^42^, ^43^ The considerable decline in transit ridership observed in Canada and worldwide throughout the pandemic^20^, ^44^ may also explain why fewer infections were observed in transit users. The reduction in transit usership was partially attributed to reduced travel demand in response to stay‐at home orders^44^ and the greater perception of SARS‐CoV‐2 transmission risk associated with public transit use.^20^ The restrictions on capacity and reduced ridership limited crowding and allowed those who continued to use public transit during the pandemic adequate space to maintain physical distancing recommendations. Similarly, the perception of increased risk may have encouraged adherence to recommended public health protocols. The light rail transit system in Ottawa was also shut down for several months during the study period for mechanical issues which may have altered transit usage. SARS‐CoV‐2 transmission risk while using public transit has been correlated with exposure time.^45^ While this occurrence was not consistently observed in this study, those who used public transit at least several times a week had higher anti‐HKU1 IgG antibody titers compared to those who did not use public transit at all (Supporting Information S1: Appendix Table 2). We were unable to assess for similar trends with HCoV‐OC43, HCoV‐NL63, and HCoV‐229E due to low incidence.

Immunocompromised individuals had reduced odds of SARS‐CoV‐2 seropositivity from natural infection at baseline and fewer CoV infections over the period of observation compared to our controls. Insufficient serological response to COVID‐19 vaccination has also been observed in solid organ transplant recipients receiving immunosuppressive agents.^46^ Disparities in antibody kinetics may explain why fewer infections were observed in the immunocompromised individuals. These individuals may develop fewer antibodies in response to natural infection or vaccination or experience rapid antibody waning. Alternatively, immunocompromised individuals and those with a higher burden of comorbidities are at greater risk of severe disease if infected by SARS‐CoV‐2 or other common respiratory viruses. These individuals may have taken more precautions during the COVID‐19 pandemic to avoid exposure such as limiting close contacts, avoiding crowds, and adhering more strictly to public health measures. As public health restrictions are removed, policymakers and healthcare professionals should be aware that this population remains at risk and may require continued special measures.

Few demographic variables were correlated with baseline IgG titers against HCoV‐HKU1, HCoV‐NL63, HCoV‐229E, and HCoV‐OC43. The high prevalence of these viruses resulted in antibody titers being consistently high among all individuals, leading to difficulties in detecting any titer differences between groups. By contrast, most of the population was naïve to SARS‐CoV‐2, enabling detection of differences in levels of exposure between demographic groups. The odds of seroprevalence were greater in those with a low household income and of ethnic minority. Our findings add to extensive evidence showing the burden of SARS‐CoV‐2 is greater among minority groups and those of low socioeconomic status.^47^, ^48^ Many factors may contribute to demonstrated inequities, such as living in more crowded households, education and finances, language or cultural barriers, and increased work in an environment that required in‐person presence during the pandemic.

The strengths of this study include well‐characterized reports of clinical and self‐reported data. We were able to analyze multiple serial blood samples for each participant with a low attrition rate over time. The combined use of serology, self‐reported PCR and RAT tests, and clinical symptoms reduce misclassification of COVID‐19 infections. Finally, the shifting landscape of public health measures and emerging variants of concern complicates the interpretation of SARS‐CoV‐2 seroprevalence in other studies. This issue was less impactful with SSO as almost all samples were collected before the arrival of the Omicron variant.

This study is subject to several limitations. The study cohort consisted of highly educated, high‐income individuals with limited ethnic diversity, which limited our ability to infer associations within lower socioeconomic groups and reduced the generalizability of results. Several factors complicate the interpretation of sCoV serology results. First, the World Health Organization does not have standardized guidelines for units, controls, or recommended thresholds when reporting sCoV serology results, so determining a threshold of infection can be challenging. Similarly, almost all individuals have detectable antibodies indicating a prior infection, so obtaining a true negative control is difficult. Evaluation of IgG and IgM titers over time were used to verify infection. However, in some cases, samples were not available from all desired time points, and the interval between available samples was too broad to detect a rise in antibody titers. For this reason, sCoV infection incidence is likely underreported and we were not always able to determine when a seasonal coronavirus infection occurred with precision. Longitudinal antibody kinetics and serologic response to infection vary by individual and are dependent on many factors including demographics, comorbidities, and disease severity.^49^ The heterogeneity in antibody response may underestimate the number of detected infections. Several participants tested positive for more than one coronavirus and one participant tested positive for all four sCoVs (Supporting Information S1: Appendix Table 4). Cross‐reactivity among CoVs is a well‐documented concern with CoV serology^10^, ^50^ and simultaneous infection for all viruses is highly improbable. Unfortunately, only one antigen was used in the serological assay and acute infection were not diagnosed by PCR, so we are unable to corroborate assay results. Future studies may consider the use of multiple antigens to improve the reliability of sCoV results. Analysis of other common respiratory viruses, including influenza and RSV in at‐risk populations, would enhance our coronavirus‐specific observations.

## 5 CONCLUSION

This analysis demonstrates that CoV incidence was reduced in immunocompromised individuals and similar between those exposed to children, transit users, and controls. This is likely a consequence of effective nonpharmaceutical public health interventions which limit viral transmission. Relaxation of these measures has resulted in a return of sCoV and other common respiratory viruses including influenza and RSV. The reappearance of seasonal respiratory viruses may have a greater impact on individuals of nonwhite ethnicity, those of low socioeconomic status, and those with an immunocompromising condition. The lasting presence of SARS‐CoV‐2 in a post‐pandemic era may continue to alter seasonal respiratory virus circulation and have consequences for population susceptibility to disease.

## AUTHOR CONTRIBUTIONS

**Aliisa Heiskanen**: Conceptualization; data curation; formal analysis; methodology; writing—original draft; writing—review and editing. **Yannick Galipeau**: Data curation; methodology; writing—review and editing. **Julian Little**: Writing—review and editing. **Marc‐André Langlois**: Writing—review and editing. **Curtis L. Cooper**: Conceptualization; formal analysis; methodology; supervision; writing—original draft; writing—review and editing.

## CONFLICT OF INTEREST STATEMENT

The authors declare no conflict of interest.

## ETHICS STATEMENT

All participants provided informed consent. Stop the Spread Ottawa study is approved by the Ottawa Health Science Network Research Ethics Board (#2020‐0481‐01H).

## Supporting information

Supporting information.

## Data Availability

The data that support the findings of this study are available from the corresponding author upon reasonable request.
