# Supplementary material for: Reduced seasonal coronavirus incidence in high‐risk population groups during the COVID‐19 pandemic
Source: Immun Inflamm Dis. 2024 Jul 18;12(7):e1342. doi: 10.1002/iid3.1342 (PMC11256882; doi:10.1002/iid3.1342)
Supplement: Supplementary file 1 — Supporting information. [file IID3-12-e1342-s001.docx]

**Appendix Table 1.** Type of test used and definition of infection for SARS-CoV-2 and seasonal coronaviruses.

| Virus | Test | Antigen | Immunoglobulin | Infection Definition | Infection Validation |
| --- | --- | --- | --- | --- | --- |
| SARS-CoV-2 | Chemiluminescent direct ELISA | S, RBD, N | IgG | SCO>1 for anti-N IgG and either anti-S or anti-RBD IgG | SCO of preceding and subsequent serum samples |
|  | PCR | N/A | N/A | Self-reported infection* | Medical records, participant follow-up |
|  | Rapid tests | N/A | N/A | Self-reported infection* | Medical records, participant follow-up |
| HCoV-NL63, HCoV-OC43, HCoV-229E,  HCoV-HKU1 | Chemiluminescent ELISA | S | IgG | 2-fold IgG titre increase between subsequent visits | Examination of IgG and IgM titre response over time |
|  | Chemiluminescent ELISA | S | IgM | Above the mean titre +(3SD/2SD) | Examination of IgG and IgM titre response over time |

*Target or cut-off information not collected for self-reported infections.

**Appendix Table 2.** Multivariate regression results measuring the magnitude of effect of increasing exposure to public transit and number of children in the household adjusted for ethnicity, sex, income, education, age, smoking status, and comorbidities. Multivariate logistic regression analysis used to assess the odds of SAR-CoV-2 seropositivity indicative of natural infection at baseline. Linear regression analysis used to assess seasonal coronavirus antibody titres at baseline. Separate models were fit for each virus.

| Demographic variable | HKU1 | | OC43 | | NL63 | | 229E | | SARS-CoV-2 | |
| --- | --- | --- | --- | --- | --- | --- | --- | --- | --- | --- |
| (reference category) | Estimate | P-value | Estimate | P-value | Estimate | P-value | Estimate | P-value | OR | 95% CI |
| Other ethnicity (v. white) | 0.06 | 0.50 | 0.17 | 0.13 | 0.05 | 0.62 | 0.19 | 0.11 | **4.55** | **1.59-13.0** |
| Female sex (v.male) | 0.04 | 0.42 | 0.02 | 0.76 | 0.04 | 0.56 | -0.04 | 0.54 | 0.74 | 0.39-1.43 |
| Income (v. +$120,000) |  |  |  |  |  |  |  |  |  |  |
| <$59,999 | 0.01 | 0.87 | 0.19 | 0.12 | 0.14 | 0.19 | -0.09 | 0.47 | 2.74 | 0.90-8.33 |
| $60,000-$89,999 | -0.10 | 0.18 | -0.01 | 0.94 | 0.02 | 0.83 | -0.03 | 0.75 | 1.81 | 0.67-4.92 |
| $90,000-$120,999 | -0.03 | 0.71 | -0.06 | 0.53 | -0.05 | 0.57 | <-0.03 | 0.98 | 0.80 | 0.31-2.05 |
| Prefer to not answer | -0.02 | 0.72 | -0.10 | 0.29 | -0.08 | 0.36 | -0.03 | 0.73 | 0.56 | 0.21-1.49 |
| Education (v.master’s) |  |  |  |  |  |  |  |  |  |  |
| High school or less | -0.14 | 0.14 | -0.16 | 0.19 | **-0.25** | **0.03** | -0.12 | 0.34 | 0.86 | 0.25-2.99 |
| Trade/college | -0.09 | 0.21 | -0.11 | 0.24 | -0.07 | 0.41 | -0.04 | 0.69 | 0.64 | 0.25-1.66 |
| Bachelor’s degree | -0.01 | 0.83 | <-0.01 | 0.99 | -0.07 | 0.36 | -0.06 | 0.48 | 1.18 | 0.52-2.67 |
| Age | <0.02 | 0.30 | <0.01 | 0.81 | <-0.01 | 0.96 | <-0.01 | 0.76 | **1.04** | **1.01-1.07** |
| Smoking (v. non-smoking) | **0.23** | **0.03** | 0.03 | 0.81 | -0.20 | 0.11 | 0.13 | 0.36 | 0.20 | 0.02-1.74 |
| Number of children* | 0.03 | 0.22 | -0.04 | 0.26 | <0.01 | 0.99 | 0.02 | 0.67 | 0.88 | 0.60-1.28 |
| Transit Use (v. never) |  |  |  |  |  |  |  |  |  |  |
| Once a week | -0.02 | 0.83 | -0.17 | 0.21 | -0.09 | 0.48 | -0.07 | 0.62 | 0.35 | 0.08-1.49 |
| A few times a week | 0.08 | 0.54 | -0.11 | 0.50 | -0.28 | 0.06 | -0.09 | 0.61 | 0.31 | 0.03-2.74 |
| Daily | **0.30** | **<0.03** | 0.04 | 0.75 | 0.11 | 0.37 | 0.08 | 0.55 | 0.27 | 0.05-1.51 |
| Number of comorbidities** | **-0.03** | **0.05** | **-0.06** | **0.01** | -0.03 | 0.10 | -0.04 | 0.08 | **0.70** | **0.56-0.89** |

*Number of children living in household

**Included cancer, diabetes, HIV, primary immunodeficiency, neurological impairment/disease, organ or bone marrow transplant, or chronic heart, lung, liver or kidney disease

**Appendix Table 3.** Sensitivity analysis removing all participants that met criteria for more than one population group of interest (n=217). Multivariate logistic regression analysis used to assess the odds of SAR-CoV-2 seropositivity indicative of natural infection at baseline. Linear regression analysis used to assess seasonal coronavirus antibody titres at baseline. Separate models were fit for each virus.

|  | HKU1 | | OC43 | | NL63 | | E229 | | SARS-CoV-2 | |
| --- | --- | --- | --- | --- | --- | --- | --- | --- | --- | --- |
| Variable | Estimate | p-value | Estimate | p-value | Estimate | p-value | Estimate | p-value | OR | 95%CI |
| Control | Ref | Ref | Ref | Ref | Ref | Ref | Ref | Ref | Ref | Ref |
| Exposed to children | <-0.01 | 0.98 | 0.02 | 0.86 | -0.03 | 0.72 | -0.01 | 0.89 | 0.89 | 0.34-2.10 |
| Transit User | <-0.02 | 0.98 | 0.03 | 0.76 | -0.04 | 0.69 | -0.08 | 0.47 | 0.45 | 0.15-1.35 |
| Immunocompromised | -0.08 | 0.25 | -0.09 | 0.34 | -0.07 | 0.46 | -0.03 | 0.75 | **0.06** | **0.02-0.20** |
| Male | Ref | Ref | Ref | Ref | Ref | Ref | Ref | Ref | Ref | Ref |
| Female | 0.03 | 0.62 | -0.02 | 0.82 | 0.02 | 0.72 | -0.02 | 0.75 | 0.50 | 0.26-1.04 |
| Age | <0.01 | 0.44 | <0.01 | 0.73 | <-0.01 | 0.99 | <-0.01 | 0.61 | 1.04 | 0.05-1.04 |
| Non-smoker | Ref | Ref | Ref | Ref | Ref | Ref | Ref | Ref | Ref | Ref |
| Smoker | **0.24** | **0.03** | -0.01 | 0.95 | -0.21 | 0.11 | 0.13 | 0.37 | 0.21 | 0.08-0.21 |
| >$120,000 | Ref | Ref | Ref | Ref | Ref | Ref | Ref | Ref | Ref | Ref |
| <$59,999 | -0.13 | 0.12 | -0.02 | 0.86 | -0.03 | 0.77 | -0.02 | 0.87 | 3.65 | 1.17-11.40 |
| $60,000-$89,999 | -0.03 | 0.75 | -0.04 | 0.74 | -0.04 | 0.69 | -0.04 | 0.74 | 0.97 | 0.33-2.90 |
| $90,000-$120,000 | -0.01 | 0.91 | -0.10 | 0.30 | -0.09 | 0.31 | -0.04 | 0.68 | 0.79 | 0.28-2.21 |
| Graduate or higher | Ref | Ref | Ref | Ref | Ref | Ref | Ref | Ref | Ref | Ref |
| Highschool or less | -0.16 | 0.10 | -0.11 | 0.39 | **-0.27** | **0.03** | -0.14 | 0.32 | 0.87 | 0.23-3.33 |
| Trade/college | -0.09 | 0.23 | -0.08 | 0.42 | -0.08 | 0.38 | -0.07 | 0.49 | 0.61 | 0.21-1.73 |
| Bachelor’s degree | -0.02 | 0.73 | 0.02 | 0.86 | -0.09 | 0.30 | -0.09 | 0.33 | 1.32 | 0.54-3.18 |
| White ethnicity | Ref | Ref | Ref | Ref | Ref | Ref | Ref | Ref | Ref | Ref |
| Other | 0.16 | 0.12 | 0.24 | 0.08 | 0.12 | 0.32 | 0.21 | 0.12 | **6.81** | **1.82-25.57** |

**Appendix Table 4.** Classification of infections for participants that tested positive for more than one coronavirus (n=7). Serology results where a participant tested positive for more than one seasonal coronavirus at a single time point was thought to be a result of cross-reactivity. It was assumed the infection was caused by the most prevalent seasonal coronavirus. Infections were classified using the hierarchal ranking system from highest to lowest expected prevalence: OC43>NL63>HKU1>229E.

| Participant |  | Positive serology result | | | | Infection classification |
| --- | --- | --- | --- | --- | --- | --- |
|  | HKU1 | OC43 | NL63 | 229E | SARS-CoV-2 |  |
| SSO0033 |  | Yes |  | Yes |  | OC43 |
| SSO0082 | Yes | Yes | Yes | Yes |  | OC43 |
| SSO0221 |  | Yes | Yes | Yes | Yes | OC43, SARS-CoV-2 |
| SSO0551 |  |  | Yes | Yes |  | NL63 |
| SSO0611 | Yes | Yes |  |  |  | OC43 |
| SSO0612 |  | Yes | Yes | Yes |  | OC43 |
| SSO0810 | Yes |  | Yes | Yes |  | NL63 |
